# Promoting Diabetes Self-Management Among Vietnamese Americans: Mixed Methods Pilot Study

**DOI:** 10.2196/80177

**Published:** 2026-05-07

**Authors:** Anna Nguyen, Valerie Eschiti, Thanh C Bui, Katherine O'Neal, Tam Nguyen, Angelina P Nguyen, Hanxia Li, Michael Machiorlatti, Kathleen Dwyer

**Affiliations:** 1 University of Oklahoma Health Campus, 1100 N. Stonewall Avenue, Oklahoma City, OK, 73117, United States, 1 4052711491 ext 49183; 2 Boston College, Chestnut Hill, MA, United States; 3 Baylor University, Dallas, TX, United States

**Keywords:** Vietnamese Americans, diabetes self-management, mobile health, mixed methods, nursing

## Abstract

**Background:**

Participating in a Diabetes Self-Management Education and Support (DSMES) program improves self-care behaviors, quality of life, and health outcomes. However, language barriers and cultural differences can hinder participation, leaving many Vietnamese Americans with limited access to DSMES services.

**Objective:**

This study aims to evaluate the feasibility, acceptability, and preliminary efficacy of a 3-month Blended Automated Links Augmented by Nurse Call and Engagement (BALANCE) intervention designed to deliver culturally tailored DSMES in the Vietnamese language, with participants monitored for 12 months afterward to assess sustained effects on key outcomes.

**Methods:**

An explanatory sequential mixed methods design was used, guided by the Practical, Robust Implementation and Sustainability Model (PRISM) framework. Feasibility and acceptability were measured by the participation rate of eligible clinics and patients, patient message response rate, and retention rate. Focus groups were conducted to assess adoption and sustainability. A pilot single-arm, prospective interventional trial was conducted with a sample of 88 Vietnamese American adults with type 2 diabetes from 10 primary care clinics. Surveys were administered at baseline and every 3 months over 12 months. Repeated measures ANOVA assessed changes in clinical outcomes at 3, 6, 9, and 12 months. Qualitative data from in-depth interviews and focus groups were thematically analyzed to validate and expand on quantitative findings. Integrated analysis using joint display enabled meta-inferences across data sources.

**Results:**

Among 88 participants (mean age 68, SD 9.8; range 35‐86 years), the intervention did not significantly affect glycated hemoglobin A_1c_ (*P*=.63) but led to a statistically and clinically significant reduction in low-density lipoprotein (*P*=.001) and improvement in exercise performance (*P*=.04). Qualitative data from 45 patient interviews reached data saturation, with 80% (n=36) describing the intervention as “convenient” and “helpful.” Clinic staff (n=18) participated in 3 focus groups and endorsed the intervention as acceptable and feasible. Mixed methods analysis confirmed high feasibility (83% clinic participation and 100% clinic retention) and acceptability (90.9% patient retention). Key barriers to sustainability included limited staffing and supply infrastructure.

**Conclusions:**

Intervention feasibility and acceptability were demonstrated but require further refinement to achieve long-term, consistent glycemic control. Findings indicated that clinic staff workload and clinic workflow were key determinants of the study’s feasibility and acceptability. Future research should test BALANCE in a fully powered randomized controlled trial to evaluate intervention effectiveness.

## Introduction

### Background

In the United States, 37.3 million people (11.3% of the population) have diabetes, with 8.25 million diabetes-related hospitalizations reported annually (327.9/1000) [1]. In 2022, the cost of diabetes in the United States was US $412.9 billion, including US $306.6 billion in direct medical costs and US $106.3 billion in indirect costs. Major contributors to indirect costs include reduced employment due to disability and lost productivity from premature deaths [2]. Diabetes is a self-managed disease, and effective management can reduce the risk of complications, morbidity, and health care costs [3,4]. Evidence indicates that participating in a Diabetes Self-Management Education and Support (DSMES) program can reduce the risk of diabetes complications and improve health outcomes [5,6].

While diabetes is the seventh leading cause of death in the overall US population, it ranks as the fifth leading cause of death among Asian Americans [7]. As members of the Asian American group, Vietnamese Americans (VNAs) face barriers to participating in existing DSMES programs due to language and cultural differences, which hinder their ability to obtain appropriate self-management information [8]. There is a limited number of Vietnamese-speaking diabetes care and education specialists, and the DSMES resources in the Vietnamese language are scarce. Language barriers often prevent effective communication with health care providers, and differences in cultural beliefs and practices may not align with standard DSMES materials. The Vietnamese community has recognized these barriers and expressed a need for formal diabetes education and support in the Vietnamese language [9].

VNAs are the fourth largest Asian American group with 90.6% of persons diagnosed with diabetes falling within the nonobese BMI range of less than 30. Nonobese VNAs have 60% higher adjusted odds of diabetes compared to nonobese non-Hispanic Whites [10]. Given the high prevalence of diabetes among VNAs, ensuring access to DSMES services is critical. The 2022 National Standards for DSMES highlight the importance of delivering services that respect cultural diversity, address social determinants of health, and leverage modern technology for engagement [5]. With mobile phone usage now nearly universal—93% of the global population has access to a mobile broadband network—mobile health (mHealth) solutions offer a powerful and accessible platform for delivering DSMES services [11].

Although DSMES programs have demonstrated effectiveness, the evidence base for interventions tailored specifically to VNAs remains limited. Prior research has primarily focused on (1) documenting culturally influenced beliefs and practices that shape diabetes self-management and help-seeking behaviors, highlighting the importance of aligning interventions with Vietnamese health beliefs and trusted information sources, and (2) emphasizing the value of community-engaged approaches to culturally tailoring DSMES content and delivery. Building on this emerging body of work, this study is novel in its implementation of a culturally adapted mHealth DSMES intervention designed to address language discordance and limited access to culturally relevant education in supporting VNAs with diabetes.

Evidence suggests that incorporating mHealth technology to provide education and support lifestyle and health behavior changes is feasible and acceptable for various populations [12-15], including Vietnamese individuals living in Vietnam [16]. However, current literature lacks evidence on the feasibility, acceptability, and effectiveness of culturally adapted interventions that leverage mHealth technology to educate and support diabetes self-management among VNAs. Hence, this study team developed a Blended Automated Links Augmented by Nurse Call and Engagement (BALANCE) intervention using mHealth technology to deliver DSMES in the Vietnamese language.

### BALANCE Intervention

BALANCE is a 3-month intervention program delivering DSMES through smartphones to empower patients in managing their diabetes by providing convenient access to knowledge, skills, and support for self-care. Following the intervention, participants were monitored for 12 months to assess sustained effects on key outcomes. The study team met with the health care providers and clinic staff at each site to discuss the referral process and operations prior to implementation. Once the clinics were ready to begin the intervention, patient education materials were distributed, and training sessions on mHealth navigation were conducted with clinic staff to prepare them for troubleshooting any patient issues with messaging. All patients received the same multicomponent intervention: (1) a printed and translated education handbook; (2) mHealth with 2-way text messaging; and (3) weekly coaching phone calls from a nurse trained in motivational interviewing techniques. These phone calls aim to reinforce the educational content, address individual challenges, and motivate patients to adhere to recommended self-care behaviors. The purpose of this study was to (1) evaluate the acceptability, feasibility, and preliminary efficacy of BALANCE and (2) assess potential factors related to BALANCE program adoption and sustainability among participating primary care clinics.

## Methods

### Study Design

This study used an explanatory sequential mixed methods research design to integrate findings of quantitative and qualitative data and to interpret results [17]. First, a pilot single-arm prospective interventional trial was conducted to assess BALANCE’s feasibility and preliminary efficacy. This phase involved collecting and analyzing data to identify intervention reach and adoption. Second, qualitative data were collected using in-depth interviews and focus groups to explore participants’ experiences and perceptions on BALANCE adoption and sustainment. Sequentially combining these methods allowed for data to be triangulated, enhanced the validity of study findings, and offered insights into the intervention’s overall impact.

### Inclusion and Exclusion Criteria

Clinics were eligible for inclusion if they served more than 10 VNA adults aged 18 years or older with a documented diagnosis of type 2 diabetes (T2D), and if clinicians and staff agreed to participate in BALANCE intervention training and anticipated completion of all study activities throughout the study period. At the patient level, individuals were included if they were 18 years of age or older; had a clinical diagnosis of T2D; were currently prescribed oral and/or injectable diabetes medications; owned a mobile phone capable of receiving text messages; and were able to read and write Vietnamese sufficiently to understand study materials and intervention content. Patients were also required to demonstrate the cognitive capacity to understand study procedures and provide informed consent. Individuals were excluded if they had moderate-to-severe cognitive impairment that limited consent capacity, were currently pregnant due to differing diabetes management requirements during gestation, lacked adequate Vietnamese literacy, or did not have reliable access to a mobile device.

### Measurements

The Practical, Robust Implementation and Sustainability Model (PRISM) was used to assess the outcomes and related contextual and environmental factors [18,19]. PRISM was developed from the Reach, Efficacy, Adoption, Implementation, Maintenance (RE-AIM) framework and hence includes all of the RE-AIM constructs and outcomes. PRISM is better because it takes into account external factors to provide a more comprehensive understanding of contextual influences on the implementation. The RE-AIM framework was applied with specific attention to patient-level reach and effectiveness, whereas the PRISM framework was used to explain the role of organizational and environmental factors in shaping clinic-level implementation and sustainability [19-23]. The types of data and data sources are summarized in Table 1.

**Table 1.** Assessments using the PRISM^a^ framework.

RE-AIM^b^ or PRISM domain	Baseline	3-month postintervention	Follow-ups	
			6 months	12 months
Patient demographics^c^	✓			
Clinic demographics^c^	✓			
Reach: absolute number of those participating in BALANCE^d^				
% of eligible clinics and patients who participated^c^	✓			
Barriers and facilitators to reach^e^	✓			
Efficacy: changes in self-care behavior, clinical laboratory results				
Changes in SDSCA^f^ scale^c^	✓	✓	✓	✓
Changes in HbA_1c_^g^, triglyceride, LDL^h^, and HDL^c^^,i^	✓	✓	✓	✓
Adoption: patient level				
Response rates to text messaging^c^	✓		✓	
% of patients enrolled at end of study period^c^	✓		✓	✓
Adoption and implementation: clinic level				
Fidelity to the study protocol and guidelines^c^		✓	✓	✓
Barriers and facilitators to implementation^e^			✓	
Sustainability: organizational infrastructure				
Sustainability of intervention at clinics^e^			✓	
Participation experiences^e^			✓	✓

a PRISM: Practical, Robust Implementation and Sustainability Model.

b RE-AIM: Reach, Efficacy, Adoption, Implementation, Maintenance.

c Programmatic data.

d BALANCE: Blended Automated Links Augmented by Nurse Call and Engagement.

e Interviews and focus groups.

f SDSCA: Summary of Diabetes Self-Care Activities.

g HbA_1c_: hemoglobin A_1c_.

h LDL: low-density lipoprotein.

i HDL: high-density lipoprotein.

#### Quantitative Survey and Clinical Outcomes

Routine programmatic data were collected to assess feasibility as well as clinical outcomes such as laboratory values and self-care behaviors. Reach was assessed using quantitative measures, including the recruitment and enrollment rates of clinics and their patients. In this study, reach was defined as the extent to which BALANCE engages with both clinics and patients. At the clinic level, reach was measured by the proportion of eligible clinics that agreed to participate in the study. At the patient level, reach was assessed by the proportion of eligible patients who enrolled in the study, relative to the total number of eligible individuals within participating clinics. To evaluate potential intervention efficacy, self-reported care behavior surveys and clinical laboratory test results were obtained at baseline, 3 months postintervention, and at 6 and 12 months. Self-care behavior changes were assessed using the Summary of Diabetes Self-Care Activities (SDSCA) scale, a translated and validated tool that measures the frequency of diabetes self-care activities over the past 7 days. The tool consists of 11 items to be rated on an 8-point Likert scale (0‐7) [24]. A higher score indicates better adherence to diet, physical activity, glucose monitoring, foot care, and medication regimen. Clinical laboratory test results were obtained from the patients’ medical records to assess the intervention’s impact on glycemic control and cardiovascular health, including hemoglobin A_1c_ (HbA_1c_), low-density lipoprotein (LDL), high-density lipoprotein (HDL), and triglycerides. Adoption was assessed by examining the acceptance and use of BALANCE intervention at both the clinic and patient levels. Implementation was assessed by looking at the fidelity of the intervention delivery, including adherence to the scheduled messages and adherence to the nurse coaching manual. In summary, 2 quantitative data sources were used, which include routine programmatic and clinical data and patient survey data. Programmatic and clinical data assessed feasibility and clinical outcomes, while survey data captured patient-reported self-care behavior outcomes.

#### Qualitative Interviews and Focus Groups

All 88 patients were invited to participate in an in-depth interview after completing the intervention. The health care providers of participating clinics were invited to participate in a separate focus group. All interviews and focus groups were conducted face-to-face with a member of the research team and a research assistant. Patient interviews were conducted in Vietnamese, and focus groups with clinic members were conducted in English. Data saturation was achieved following the completion of 45 patient interviews, with 5 to 10 patients from each clinic. Saturation was determined when no new themes, insights, or patterns emerged from successive interviews, indicating that additional data collection was unlikely to yield novel information relevant to the study objectives [25]. Three focus groups were conducted with 6 physicians, 1 physician assistant, and 11 clinic staff (1 to 3 per clinic). Interviews and focus groups were audio-taped, and a trained research assistant took field notes as a summary. The in-depth interview questions and focus group questions were designed to gather information related to their participation experiences.

### Data Analysis

#### Quantitative Data

Descriptive statistics were used to summarize baseline and follow-up measurements for clinical laboratory tests and self-care behavior surveys. Repeated measures 1-way ANOVA was used to explore treatment effects at 3-, 6-, 9-, and 12-month postintervention. All analyses were conducted using Python (version 3.11.13; Python Software Foundation). A 2-sided *P *value of <.05 was used as the threshold for statistical significance.

#### Qualitative Data

Patient interview recordings were translated from Vietnamese to English before being transcribed. All recordings were transcribed and uploaded to NVivo 15 software program (QSR International) for organizing and categorizing themes. Direct content analysis was used to assess technical implementation, participant engagement, cultural relevance, language appropriateness, and BALANCE’s impact on health behaviors and clinical outcomes. Direct content analysis is a structured method used to identify key concepts and is appropriate to use for analyzing predefined categories or themes [26]. This method provided valuable insights into feasibility and acceptability, as well as information that can inform the refinement and optimization of future intervention updates. Transcripts were independently coded by 2 study investigators, with differences in coding resolved via discussion. The 2 coders then identified themes and relationships among finalized codes.

### Ethical Considerations

Eligible individuals went through an informed consent process to receive comprehensive information about the study, including its purpose, procedures, potential benefits, and risks. During this session, a member of the study team explained the study in detail, answered any questions, and ensured that those who are eligible fully understood what their involvement would entail. Confidentiality and data protection were also explained. Participants were assured that their personal information would be kept secure and used only for research purposes. Those interested in participating provided a written consent to participate. Before any data collection began, the study received approval from the University of Oklahoma Health Sciences Institutional Review Board (IRB 14700), ensuring that all ethical standards and guidelines were met. Study participants received US $50 in the form of a gift card as compensation for their time and effort at each of the data collecting points.

## Results

### Demographics and Baseline Characteristics

#### Clinic Level

Ten primary care clinics participated in the study. Among these, 4 clinics were independently owned and operated by a single physician, while the remaining 6 were part of larger health systems with multiple providers. All clinics, with the exception of one that accepted only private insurance, offered services to patients covered by Medicare, Medicaid, and private insurance plans. The collaborative network with primary care providers played a crucial role in integrating BALANCE into routine health care delivery. Their participation facilitated a seamless incorporation of the intervention into existing clinical workflows, supporting efficient implementation and enhancing the potential for broader adoption across varied primary care environments.

#### Patient Level

The study enrolled 88 adults with T2D from primary care clinics in the Central United States. Participants ranged in age from 35 to 86 years, with an average age of 68 (SD 9.8) years. Only 5% of participants (4/88) had ever attended at least one DSMES session since the time of their diagnosis, and none had completed the program. Regarding health behaviors and comorbid conditions, 5% (4/88) were current smokers, 70% (62/88) were taking hypertension medication, and 77% (68/88) were taking medication for hyperlipidemia. Within the first week of the intervention, 5% (4/88) of participants reported difficulty reading text messages or printed materials due to vision limitations. All 4 were given the option to remain in the study, as they had live-in family members who could assist them. Refer to Table 2 for a detailed breakdown of patient demographic characteristics.

**Table 2.** Demographic characteristics of patients.

Characteristics	Total
Gender, n (%)	
Men	52 (59)
Women	36 (41)
Years with diabetes, n (%)	
0‐1	8 (9)
2‐3	12 (14)
4‐5	12 (14)
6‐7	9 (10)
8‐9	5 (6)
>9	42 (48)
Attended some DSMES^a^ since diagnosis, n (%)	
Yes	4 (5)
No	84 (96)
Years residing in the United States, n (%)	
0‐5	8 (9)
6‐10	4 (5)
>10	76 (86)
Received family support for diabetes care, n (%)	
Yes	39 (44)
No	49 (56)

a DSMES: Diabetes Self-Management Education and Support.

### Feasibility and Acceptability Outcomes

#### Intervention Reach

To contextualize intervention reach, the clinic-level recruitment and enrollment are considered indicators of strong engagement with the primary care practice clinicians. Of the 10 eligible clinics identified, 10 (100%) agreed to participate in the study. Across these clinics, 175 eligible patients were identified and were invited to enroll. Of those invited, 88 consented to participate, resulting in a 50% patient enrollment rate. This level of participation demonstrates moderate patient-level reach, indicating feasibility and general receptiveness to the research intervention study.

#### Adoption and Implementation at the Clinic Level

The 3 focus groups comprised 60% males and represented various clinic roles, including physicians (n=6), physician assistants (n=1), registered nurses/clinic managers (n=2), licensed practical nurses (n=4), and medical assistants (n=5). Clinicians emphasized the importance of cultural tailoring and translating materials into Vietnamese, noting that these aspects significantly enhanced patient comprehension of their treatment regimen.

Providing education in the Vietnamese language is crucial given the unique cultural and linguistic barriers that many Vietnamese patients face in managing type 2 diabetes.

[Physician, P03]

Many patients struggle with understanding the complexities of diabetes management due to a lack of linguistically appropriate resources.

[Physician, P04]

This is a much-needed intervention to improve health outcomes and bridge the access gap.

[Clinic staff member, RN1]

The integration of mHealth technologies was praised for its relevance in meeting the needs of those without transportation. Physicians reported that their patients found the 2-way text messaging system and weekly nurse coaching calls beneficial for answering questions related to daily self-care activities. Additionally, motivational interviewing techniques used during nurse coaching calls were seen as particularly valuable in fostering behavior change. Three physicians (P01, P02, and P06) noted that the coaching calls added a personalized element that encouraged patients to be more engaged in their care. They noted that patients were more likely to set realistic goals and take actionable steps toward improving their diabetes management.

#### Adoption and Sustainment at the Patient Level

Among study participants, 89.7% (79/88) engaged with at least 2 of the 3 BALANCE intervention components. All individuals who participated in the intervention completed the postintervention self-care behavior survey. Additionally, 74% (65/88) attended one or more scheduled nurse coaching calls, indicating a strong interest in personalized support. These qualitative data were derived from the patient survey dataset and metrics extracted from the mHealth program database. Qualitative feedback from in-depth interviews with patients (n=45) highlights the acceptability, revealing high satisfaction with the intervention, particularly the ease of navigating the mHealth messages. Patients reported feeling more empowered and confident in managing their diabetes and appreciated the culturally relevant education materials. All patients valued the convenience of receiving education and support through their mobile phones, eliminating the need to travel to in-person classes, which contributed to their decision to complete the study.

I like the way messages are sent on a daily basis, just short and simple to understand. I also like that we can read more details in the book. And with it being in Vietnamese and showing Vietnamese food was exactly what we needed.

[Female, 65 years, participant]

I don’t have time to drive to class and sit there for hours. Receiving text messages on my phone made it easy to learn and be informed, even with my busy schedule. This made my involvement easier.

[Female, 67 years, participant]

An analysis of weekly response rates to mHealth messaging revealed a gradual decline in engagement over the 3-month period. Patients were classified as low users if they responded to 2 or fewer messages per week, and as high users if they responded to 3 or more. In week 1, there were 24 low users, increasing steadily to 40 by week 12. These trends indicate a shift toward lower engagement, with fewer patients sustaining high levels of message interaction over time.

Through individual interviews, some patients expressed a desire for more interactive features within the intervention, such as quiz questions, to further enhance their engagement and learning experience. Others expressed that mHealth text messaging was unsuitable due to their limited data phone plans. The daily text messages and videos required more data than provided on some patients’ phone plans have available, potentially leading to extra charges if data limits were exceeded and making the intervention financially burdensome. Conversely, a few patients suggested extending the duration of the intervention to provide ongoing support and reinforcement.

### Organizational Infrastructure for Intervention Sustainability

Through focus groups, clinic members were asked to reflect on their experiences related to implementation and the need for sustaining BALANCE over time. A consistent theme in all focus groups was the need to integrate the DSMES service into their routine workflow, including streamlining processes to reduce administrative burdens and decrease the workload for clinic staff.

We see the value in this program, but to keep it going, it has to be woven into our daily workflow. Right now the onboarding process feels heavy, and simplifying it would be best.

[Physician, P03].

In addition to streamlining the clinic workflow, 4 key components were identified as essential for intervention sustainability: executive leadership involvement, integration of operating procedures, personnel, and supplies for patient home testing and monitoring.

Executive leaders, including health system and clinic directors, play a crucial role in setting the vision and strategic direction for sustainability initiatives. Their buy-in enhances advocacy and support, ensuring the integration of BALANCE into clinics and the establishment of policies to embed the intervention into routine operations. Organizational leaders, including clinic managers, can also engage and empower staff by appointing personnel responsible for overseeing and driving intervention sustainability initiatives. Additionally, leadership administrators can ensure the sustainability of necessary equipment by budgeting and sourcing supplies to provide patients with the materials they need for home testing and monitoring. Many participants voiced unmet needs for glucose meters, test strips, and related home-monitoring supplies. In response to these patient-identified needs, clinics should finance glucose meters, test strips, and other testing supplies at the clinic level when patients’ insurance does not provide coverage, particularly as DSMES becomes integrated into primary care clinic services. Future implementation addressing these 4 components can contribute to an infrastructure that supports sustainable practices and ensures that incorporating BALANCE is not just an add-on but an integral part of the organization’s practice and operations.

### Preliminary Efficacy

Patient outcomes on HbA_1c_ levels did not show a statistically significant change (*P*=.63). Similarly, triglyceride levels decreased from 156.2 mg/dL to 138.1 mg/dL, though not statistically significantly (*P*=.17). The LDL levels statistically significantly decreased from 77.2 mg/dL to 68 mg/dL (*P*=.001), indicating a clinically positive impact. The HDL levels slightly decreased from 49.9 mg/dL to 47.1 mg/dL, but this was not statistically significant (*P*=.15). Diabetes self-care behaviors measured by the SDSCA improved by over 1 point from baseline to 12 months, with exercise performance showing statistical significance (*P*=.04). Overall, the intervention significantly improved LDL levels and showed positive trends in triglyceride levels and self-care behaviors, suggesting potential efficacy. Refer to Table 3 for detailed outcomes of estimated 12-month differences and Table 4 for detailed analysis of specific self-care behaviors.

**Table 3.** Changes in clinical laboratory tests and self-care behavior outcomes.

Health measure	Baseline, mean (SD)	3 months, mean (SD)	6 months, mean (SD)	9 months, mean (SD)	12 months, mean (SD)	Change (baseline to 12 months; 95% CI)	*P* value
HbA_1c_^a^	7.1 (1.4)	7.1 (1.4)	7.2 (1.1)	7.2 (1.1)	7.2 (0.8)	0.01 (−0.54 to 0.56)	.63
Triglyceride	156.2 (81.9)	139.7 (66.3)	177 (116.6)	157.2 (71.2)	138.1 (69.4)	−22.79 (−55.51 to 9.93)	.17
LDL^b^	77.2 (29.2)	76.4 (29.5)	84.5 (36.7)	69.5 (26.7)	68 (25)	−19.85 (−34.43 to −5.27)	.001
HDL^c^	49.9 (11.6)	50 (12.2)	49.7 (17.9)	47.7 (10.6)	47.1 (12.7)	−3.9 (−8.2 to 0.4)	.15
SDSCA^d^	4.4 (1.5)	5.0 (1.3)	5.0 (1.1)	5.1 (1.2)	5.5 (1.0)	0.38 (−0.58 to 1.34)	.40

a HbA_1c_: hemoglobin A_1c_.

b LDL: low-density lipoprotein.

c HDL: high-density lipoprotein.

d Diabetes Self-Care Behaviors measured by the Summary of Diabetes Self-Care Activities (SDSCA). Score ranges 0‐7 with higher score indicating better adherence to diabetes self-care behaviors.

**Table 4.** Detailed analysis of specific self-care behaviors of the SDSCA^a^.

Diabetes self-care activities	*F* test (*df*)	*P* value
Diet	0.74 (4)	.41
Exercise	5.18 (4)	.04
Glucose monitor	3.97 (4)	.07
Foot care	2.07 (4)	.18
Smoking	1.00 (4)	.34

a SDSCA: Summary of Diabetes Self-Care Activities.

The overarching mixed methods domain from integrating the quantitative and qualitative data is reach, efficacy, adoption at the patient level, potential factors related to adoption, and organizational infrastructure for sustainability. Quantitative findings aligned with qualitative results, indicating strong support of BALANCE from both providers and patients, who believed it to be beneficial for the VNAs. The meta-inferences at the patient level are presented in Table 5 and at the clinic level are presented in Table 6. The survey results reflect the entire study sample, whereas the quotations are drawn from a subset of participants, specifically patients who took part in interviews and providers who joined focus groups.

**Table 5.** Joint display results of RE-AIM^a^ implementation outcomes at the patient level.

Quantitative data summary	Qualitative data summary	Exemplar quote	Meta-inference and interpretations
Reach			
10/10 (100%) of eligible clinics participated 88/175 (50%) of eligible patients participated Age: 34‐86 years (mean 67)	The sample had a higher percentage of people who were older (≥65 years) compared to the general population in the geographic area. This is the first diabetes education program offered in the Vietnamese language that leverages mHealth^b^ technology and thus it was well received.	“This program is helpful for old people like me who can’t read or understand English.” (Patient, 72 years) “Most Vietnamese patients declined referrals to a DSMES program offered in English, or they came to one session and never came back.” (Physician, 57 years)	Most patients were willing to participate in BALANCE^c^ because it is available in the Vietnamese language and coincides with their primary care visit rather than coming to separate in-person health education visits.
Efficacy			
Outcomes: changes from baseline to 12 months, 95% CI (*P* value) HbA_1c_^d^ (*P*=.63) Triglyceride (*P*=.17) LDL^e^ (*P*=.001) HDL^f^ (*P*=.15) SDSCA^g^ (*P*=.40)	Patients recognized the importance of changing self-care behaviors. However, many patients had a relapse after the text messaging stopped as some patients felt they were “no longer being watched.” Among patients who had improved laboratory test results after completing the intervention, many felt a sense of achievement and then relapsed.	“I used to think that avoiding sweet food was enough to control my sugar, but I know better now.” (Patient, 67 years) “I eat less rice, and I take my medicines daily now. My blood sugar in the morning has been more stable since I paid attention to what I ate for dinner the night before.” (Patient, 56 years)	BALANCE can potentially enhance diabetes-related knowledge, promote health behavior changes, and improve glycemic control. While HbA_1c_ changes were not statistically significant, there was a significant reduction in LDL and improvement in exercise performance.
Adoption at the patient level			
79/88 (89.7%) of patients adopted the intervention while 80/88 (90.9%) completed the study (adoption was defined as actively engaged in at least 2 of 3 intervention components).	Facilitators to the intervention adoption at the patient level include the perceived helpfulness and comprehensibility, appropriate mode of delivery, and ease of use. Main barriers to the intervention adoption were the large consumption of phone data for videos and the inability to adopt a new lifestyle because of family responsibilities.	“The videos are helpful; the messages are nice and short and easy to read*.”* (Patient, 61 years) “I skipped a few days with videos because it was using up too much of my data.” (Patient, 57 years) “Knowledge is good, but I can’t think about diabetes every day; I have to work and take care of my grandchildren.” (Patient, 67 years)	Although some patients encountered challenges with viewing text messages/videos, reading printed workbook and/or following the recommended self-care behavior change, there were positive perceptions and experiences, perceived benefits, and willingness to continue with the intervention.

a RE-AIM: Reach, Efficacy, Adoption, Implementation, Maintenance.

b mHealth: mobile health.

c BALANCE: Blended Automated Links Augmented by Nurse Call and Engagement.

d HbA_1c_: hemoglobin A_1c_.

e LDL: low-density lipoprotein.

f HDL: high-density lipoprotein.

g SDSCA: Summary of Diabetes Self-Care Activities.

**Table 6.** Joint display results of PRISM^a^ implementation outcomes at the clinic level.

Quantitative data summary	Qualitative data summary	Exemplar quote	Meta-inference and interpretations
Potential factors related to BALANCE^b^ adoption			
Clinics that are independently owned were quicker in initiating the intervention Clinics that operate within a large health system had a delay in initiating the intervention because of multiple layers in the approval process	All clinic staff and clinicians perceived the intervention as beneficial as long as the clinic staff’s workload in implementing the intervention is minimal. Complex referral process and lengthy program enrollment process were perceived as a burden for clinic staff.	“This program is crucial given the unique barriers that Vietnamese patients face with self-management, but it needs to fit into our workflow.” (Physician) “Transportation have been an issue for our patients with the in-person classes, so text messaging is a great strategy.” (Physician)	The need for culturally and linguistically appropriate DSMES^c^ was clearly recognized by the providers. However, approvals from health system administrators can influence intervention support and referrals are based on the simplicity of program integration into existing workflow.
Organizational infrastructure for BALANCE sustainability			
Four components were identified as necessary for sustainment: Buy-in from executive leadership Program integration with policies and operating procedures Personnel Supplies (blood pressure monitors, glucometers, and strips)	The enrollment process takes approximately 30 minutes per patient: complete the consent, demographics, and baseline data. Maintaining this intervention will require either a member of the research team to be present at the clinic or to embed the task in the job description of clinic staff.	“Implementing it doesn’t take much time and effort from our clinic staff once patients are enrolled.” (Physician) “It would be ideal for our EPIC system to integrate with your system to trigger referrals for the program and you take it from there.” (Physician)	Providers and clinic staff suggested dedicated personnel, a dedicated day of the week for intervention enrollment, and electronic health records to be interfaced. Another suggestion is to approach large health systems starting at the executive level administrators.

a PRISM: Practical, Robust Implementation and Sustainability Model.

b BALANCE: Blended Automated Links Augmented by Nurse Call and Engagement.

c DSMES: Diabetes Self-Management Education and Support.

## Discussion

### Principal Findings

The findings from this pilot study indicate that the multicomponent and culturally tailored BALANCE intervention is feasible and acceptable among VNA participants. Out of 175 eligible patients invited to participate in the study, 88 consented, resulting in a 50% enrollment rate. Although this is a lower rate compared to the general population, it is considered to be acceptable, as participation rates in clinical trials among Asian Americans are notably lower compared to other racial and ethnic groups. Studies have shown that Asian Americans are less willing to participate in health research than African Americans, Hispanics or Latinos, and Caucasians [27]. For instance, Asian Americans’ participation in clinical trials ranges from 0.75% in cardiovascular trials to 4% in dermatologic trials [28]. This is significantly lower than the participation rates of other groups, such as African Americans, who can account for up to 45% in psychiatric trials [28]. A 50% participation rate and a 90% adoption rate support its feasibility and acceptability. Patient interviews further validated the cultural relevance of the educational materials, which enhanced engagement and contributed to a 100% study completion rate.

Both clinicians and patients reported multiple advantages of BALANCE compared to traditional in-person classes, including eliminating transportation needs, the integration of cultural content, bilingual support, and easy-to-navigate messages. While most patients preferred and appreciated the short daily messages, some reported that the 3-month daily texts with multiple reminders contributed to message fatigue. These findings align with other mHealth intervention research suggesting that simplicity and brevity should be key features of phone-based text messaging design [29,30]. The decline in engagement with mHealth messaging over time is a common trend in digital health interventions. Reasons include message fatigue, reduced perceived relevance, and lack of personalization, which can cause participants to lose interest [31].

Conversely, many patients preferred ongoing messages beyond the 3-month period to maintain engagement and motivation for self-care behavior change, while clinicians suggested expanding BALANCE to support self-management of other chronic conditions, such as hypertension and hyperlipidemia. This underscores the importance of considering individual patient circumstances, such as data availability, when designing mHealth messages. As initial enthusiasm fades, users may deprioritize the messages, especially if they feel repetitive or disconnected from their current needs. Future refinement of BALANCE will include personalizing content, varying message formats, and adding interactive or gamified elements to help sustain interest and encourage continued participation [31,32]. In future work related to this study, a linear mixed-effects model will be used to examine the association between outcome variables and the level of adoption, categorized as high (3 or more engagements) versus low (2 or fewer engagements).

### Comparison With Prior Work

In contrast to previous studies where culturally adapted DSMES interventions often showed inconsistent effects on physical activity and participant engagement, these findings highlight statistically significant improvements in exercise behavior and self-management perceptions among BALANCE participants. Although the overall SDSCA score did not reach significance (*P*=.40), the intervention improved exercise performance, which showed a statistically significant change over time (*P*=.04). Furthermore, data from in-depth interviews and focus groups revealed satisfaction with BALANCE where participants regarded it as a positive change in diabetes self-management behaviors. These findings suggest that culturally tailored DSMES interventions can effectively improve self-care behavior and clinical outcomes among study participants but will need additional nudging to maintain desired outcomes. Future refinements will also need to be focused on connecting the relationship between intentions, attitudes, and perceived behavior control.

Our mixed methods analysis revealed 2 significant insights. First, the study team observed a strong connection between culturally tailored DSMES and its acceptance by both clinicians and patients, highlighting how culturally relevant content can enhance engagement and adherence to self-management recommendations. Second, several barriers were identified that are related to the sustainability of BALANCE, including restricted phone data plans and limited resources such as clinic personnel and glucose testing supplies. The need for ongoing commitment and support posed suitable opportunities to maintain the intervention’s adoption and sustainment.

Nonadherence to treatment regimen and self-modification of prescribed diabetes medications emerged in in-depth interviews. Specifically, some patients reported reducing their dosage to half of the prescribed amount upon perceiving their blood glucose levels to be within a “normal” range. Cultural beliefs may explain this, as few patients (5%) reported ever receiving DSMES. Evidence indicates that nonadherence to prescribed treatments may lead to poorer diabetes control and increased risk of hospitalization and mortality [33,34]. As a result, diabetes illiteracy exacerbates health disparities among many VNA patients with limited resources for effective self-management. Although BALANCE eliminated the language barriers and improved DSMES access by leveraging mHealth technology, the study team recognized that correcting certain cultural beliefs will be a gradual process.

The HbA_1c_ change was not statistically significant at the 3-month period following the intervention and was slightly increased at the 12-month measurement. It should be noted that the baseline mean HbA_1c_ was 7.1 (SD 1.4), which is very close to the 2025 Standards of Care in Diabetes recommends an A_1c_ goal of less than 7% for most nonpregnant adults [35]. It will be important to assess the efficacy of this intervention in VNAs with poor glycemic control or those with HbA_1c_ higher than 7.5%. Furthermore, the 2025 Standards of Care in Diabetes recommends an LDL cholesterol goal of less than 70 mg/dL for people with diabetes aged 40 to 75 years with higher cardiovascular risk [36]. Participants in this study achieved a mean LDL within the recommended goal range at the 9-month measurement, which was sustained in the 12-month measurement. Lowering LDL levels can reduce diabetes complications such as heart disease, stroke, and kidney disease.

### Strengths and Limitations

This study has several strengths, including a mixed methods design integrating programmatic data with in-depth interviews, the first culturally tailored mHealth DSMES intervention for VNAs, and the use of a potentially scalable and accessible mHealth delivery approach. However, this study has some limitations. First, the small sample size makes it challenging to detect statistically significant differences between pre- and postintervention, and a single geographic location limits the generalizability of the findings. However, given the pilot and feasibility nature of this study, these issues are justifiable. Indeed, we demonstrated that it was highly feasible to obtain quantitative RE-AIM and clinical outcomes in this study, which warrants a future fully-powered randomized controlled trial (RCT) to evaluate the BALANCE’s efficacy and its implementation. Second, most participants were first-generation immigrants and aged 65 years or older, so the applicability of BALANCE to second-generation immigrants or younger individuals remains to be verified. Third, we did not investigate which education component was most effective at improving outcomes. Future RCTs with a more complex, multiple-assignment design may be needed to address this issue.

### Conclusions

The pilot study demonstrated the feasibility, acceptability, and preliminary efficacy of a culturally tailored 3-month intervention using mHealth technologies. Qualitative data complemented the quantitative results, showing that clinic members and patients were overwhelmingly supportive of BALANCE and perceived many benefits. Future research should rigorously evaluate the BALANCE intervention’s effectiveness and implementation outcomes through a fully powered RCT. Furthermore, this study supports the potential for wider implementation and scaling of this intervention to improve DSMES access among VNAs and explores long-term sustainability and scalability in different population groups who face similar language and cultural barriers.

## Abbreviations

BALANCE: Blended Automated Links Augmented by Nurse Call and Engagement
DSMES: Diabetes Self-Management Education and Support
HbA_1c_: hemoglobin A_1c_
HDL: high-density lipoprotein
LDL: low-density lipoprotein
mHealth: mobile health
PRISM: Practical, Robust Implementation and Sustainability Model
RCT: randomized controlled trial
RE-AIM: Reach, Efficacy, Adoption, Implementation, Maintenance
SDSCA: Summary of Diabetes Self-Care Activities
T2D: type 2 diabetes
VNA: Vietnamese American
